# High Fructose Causes More Prominent Liver Steatohepatitis with Leaky Gut Similar to High Glucose Administration in Mice and Attenuation by *Lactiplantibacillus plantarum* dfa1

**DOI:** 10.3390/nu15061462

**Published:** 2023-03-17

**Authors:** Thunnicha Ondee, Krit Pongpirul, Kanyarat Udompornpitak, Warumphon Sukkummee, Thanapat Lertmongkolaksorn, Sayamon Senaprom, Asada Leelahavanichkul

**Affiliations:** 1 Department of Preventive and Social Medicine, Faculty of Medicine, Chulalongkorn University, Bangkok 10330, Thailand; 2 School of Global Health, Faculty of Medicine, Chulalongkorn University, Bangkok 10330, Thailand; 3 Department of International Health, Johns Hopkins Bloomberg School of Public Health, Baltimore, MD 21205, USA; 4 Clinical Research Center, Bumrungrad International Hospital, Bangkok 10110, Thailand; 5 Department of Infection Biology & Microbiomes, Faculty of Health and Life Sciences, University of Liverpool, Liverpool L69 3GB, UK; 6 Department of Microbiology, Faculty of Medicine, Chulalongkorn University, Bangkok 10330, Thailand; 7 Center of Excellence in Clinical Pharmacokinetics and Pharmacogenomics, Department of Pharmacology, Faculty of Medicine Chulalongkorn University, Bangkok 10330, Thailand; 8 Research Management and Development Division, Office of the President, Mahidol University, Nakhon Pathom 73170, Thailand; 9 Center of Excellence in Translational Research in Inflammation and Immunology Research Unit (CETRII), Department of Microbiology, Chulalongkorn University, Bangkok 10330, Thailand; 10 Nephrology Unit, Department of Medicine, Faculty of Medicine, Chulalongkorn University, Bangkok 10330, Thailand

**Keywords:** glucose, fructose, obesity, prediabetes, *Lactiplantibacillus plantarum*

## Abstract

High-sugar diet-induced prediabetes and obesity are a global current problem that can be the result of glucose or fructose. However, a head-to-head comparison between both sugars on health impact is still lacking, and *Lactiplantibacillus plantarum* dfa1 has never been tested, and has recently been isolated from healthy volunteers. The mice were administered with the high glucose or fructose preparation in standard mouse chaw with or without *L. plantarum* dfa1 gavage, on alternate days, and in vitro experiments were performed using enterocyte cell lines (Caco2) and hepatocytes (HepG2). After 12 weeks of experiments, both glucose and fructose induced a similar severity of obesity (weight gain, lipid profiles, and fat deposition at several sites) and prediabetes condition (fasting glucose, insulin, oral glucose tolerance test, and Homeostatic Model Assessment for Insulin Resistance (HOMA score)). However, fructose administration induced more severe liver damage (serum alanine transaminase, liver weight, histology score, fat components, and oxidative stress) than the glucose group, while glucose caused more prominent intestinal permeability damage (FITC-dextran assay) and serum cytokines (TNF-α, IL-6, and IL-10) compared to the fructose group. Interestingly, all of these parameters were attenuated by *L. plantarum* dfa1 administration. Because there was a subtle change in the analysis of the fecal microbiome of mice with glucose or fructose administration compared to control mice, the probiotics altered only some microbiome parameters (Chao1 and *Lactobacilli* abundance). For in vitro experiments, glucose induced more damage to high-dose lipopolysaccharide (LPS) (1 µg/mL) to enterocytes (Caco2 cell) than fructose, as indicated by transepithelial electrical resistance (TEER), supernatant cytokines (TNF-α and IL-8), and glycolysis capacity (by extracellular flux analysis). Meanwhile, both glucose and fructose similarly facilitated LPS injury in hepatocytes (HepG2 cell) as evaluated by supernatant cytokines (TNF-α, IL-6, and IL-10) and extracellular flux analysis. In conclusion, glucose possibly induced a more severe intestinal injury (perhaps due to LPS-glucose synergy) and fructose caused a more prominent liver injury (possibly due to liver fructose metabolism), despite a similar effect on obesity and prediabetes. Prevention of obesity and prediabetes with probiotics was encouraged.

## 1. Introduction

One of the main global health problems is obesity-induced diabetes mellitus, which is linked to other problems, including dyslipidemia and cardiovascular disease, with devastating consequences, especially in critically ill patients [1]. High carbohydrate intake is one of the important causes of metabolic syndrome that induces obesity and is diagnosed by three of the following five criteria; impaired fasting blood glucose (FBG), high fasting blood triglyceride, low high-density lipoprotein (HDL), high blood pressure, and abnormal waist circumference (apple-shaped body) [2]. Interestingly, metabolic syndrome is found in approximately 20–25% of the world’s population, and impaired fasting blood glucose can progress to a prediabetes condition (higher plasma glucose than normal, but not high enough for a diabetes diagnosis) or open type 2 diabetes mellites that are associated in part with an unhealthy diet [3]. Among high-carbohydrate diets, glucose is well known for its adverse effects, while fructose appears to have fewer health effects, as fructose syrup, but not glucose or sucrose, is currently used for most beverages and soft drinks [4]. There are inconsistent data on the impacts of fructose on body weight, as some publications do not demonstrate obesity after fructose administration in rodents [5,6,7], while other groups report weight gain from fructose [8]. On the one hand, some publications indicate that fructose does not exert specific metabolic effects on increased body weight [9]. However, fructose may be a carbohydrate with greater obesogenic potential than other sugars, in part due to the increased accumulation of triacylglycerol in hepatocytes [10]. Fructose and glucose seem to have some differences in liver lipogenesis and insulin signaling [11]; however, again with some controversial data [12]. Likewise, glucose has been mentioned to have more adverse effects on enterocytes and gut microbiota than fructose [13], but the data on this topic are still very limited. In fact, obesity-induced inflammation through several mechanisms, such as hypoxic hypertrophic adipocyte, adipocyte apoptosis [14,15], reduced adiponectin with elevation of leptin [16], mitochondrial dysfunction [17], and metabolic endotoxemia due to intestinal barrier defect [18] leads to atherosclerosis, a major vascular consequence of obesity [19]. In fact, endotoxin (lipopolysaccharide; LPS) has a molecular weight of 10–100 kDa and is found in the cell walls of Gram-negative bacteria, which are the most abundant organisms in the gut microbiota [20]. Although molecules with molecular weight (MW) greater than 0.6 kDa are generally unable to cross the tight junction barrier of the intestinal tract under normal circumstances [20], intestinal damages severe enough to allow translocation of pathogen molecules from the intestinal tract into the bloodstream are often called ‘leaky intestinal or intestinal leakage’ in several conditions, including obesity and diabetes [21,22,23,24]. Immune responses against endotoxins during obesity can be very potent because the activation by pathogen-associated molecular patterns (PAMP) of the organism is naturally more severe than the response toward damage-associated molecular patterns (DAMP) of the host cell [25]. Perhaps, glucose consumption could directly induce intestinal inflammation leading to a defect of the gut barrier and metabolic endotoxemia with systemic inflammation [26]. However, fructose could induce more potent steatohepatitis, known as nonalcoholic fatty liver disease (NAFLD) or obesity-induced steatohepatitis, which is more profoundly exacerbated by the presence of LPS in the blood circulation [27]. Therefore, the comparison between glucose and fructose consumption in terms of obesity, prediabetes, leaky gut, and systemic inflammation is interesting. 

The high abundance of carbohydrates in the diet produces gut dysbiosis [28] (an alteration of organisms in the intestinal tract [29]), in part due to the different abilities in carbohydrate metabolism between different groups of bacteria [30,31]. The increase in gut mucosal damage means that high MW molecules, such as LPS, can be directly translocated into the liver and circulatory system [32,33] leading to more severe steatohepatitis and systemic inflammation, respectively. Because intestinal leakage in several causes [32] is mainly attenuated by host-beneficial probiotics [34,35,36], in part through improved intestinal integrity by some anti-inflammatory substances [37,38,39,40,41]. Among several strains of probiotics, *Lactiplantibacillus plantarum species* (previously known as *Lactobacillus plantarum*) are lactic acid producing bacteria that are often used [42], in part due to (i) the tolerance to acid in the stomach and bile in the intestine [43], (ii) the well-known synergy with other combinations of probiotics [44], and (iii) the relatively easy preparation procedure. Furthermore, the adverse effect of *Lactobacilli* probiotics is not prominent and is reported primarily in immune-compromised hosts [45]. Recently, *L. plantarum* dfa1 isolated from the Thai population demonstrated probiotic properties in vitro [46]. Furthermore, Thai isolated probiotics may have some properties different from Caucasians isolated probiotics due to the possible influence of some specific characteristics (ethnics, diets, climate, and co-evolution impact) in the population [47,48,49]. Then we hypothesized that (i) there could be differences between high glucose and high-fructose diets and (ii) *L. plantarum* dfa1 could attenuate the conditions of mice with a high-carbohydrate diet. Therefore, the administration of a high-sugar diet in mice with or without *L. plantarum* dfa1 was compared with in vitro experiments.

## 2. Materials and Methods

### 2.1. Animals and Animal Model

The animal care and use procedure was authorized by the Faculty of Medicine of Chulalongkorn University, Bangkok, Thailand (SST 025/2563) according to the standards of the US National Institutes of Health. Then, 8-week-old male C57BL/6 mice were purchased from Nomura Siam (Pathumwan, Thailand). Mice with regular diet (RD) using standard laboratory food (Mouse Feed Food No.082, C.P. Company, Bangkok, Thailand) consisting of 55.5% carbohydrates (no sugar), 31.3 % protein, and 13.2% fat with an energy content calculated at 3.04 kcal/g. The high-carbohydrate diet was modified regular mouse food (23.5% protein and 10.0% fat) with 66.5% carbohydrates using 24.8% glucose or 24.8% fructose for the high-glucose diet (HGD) and the high-fructose diet (HFrD), respectively, with an energy content calculated at 3.04 kcal/g that is equal to the energy of the regular diet. *Lactiplantibacillus plantarum* dfa1 was isolated from the feces of Thai volunteers from the Thailand Science Research and Innovation Research Institute (TSRI: RDG6150124) at the Chulalongkorn University Faculty of Medicine [26]. The bacteria stock culture was stored in deMan Rogosa Sharpe broth (MRS) (Oxoid, Hampshire, UK) containing 20% (*vol*/*vol*) glycerol at −80 °C and cultured on MRS agar under anaerobic conditions using gas generation sachets (Anaero Pack-Anaero, Mitsubishi Gas Chemical, Tokyo, Japan) at 37 °C for 48 h before use. The spectrophotometer (Bio-Rad, Smart Spec 3000; Bio-Rad, Hercules, CA, USA) at optical density using a 600 nm wavelength (OD600) of 0.15 (approximately 1 × 10^9^ CFU) in 0.5 mL of phosphate buffer solution (PBS) or PBS alone was administered orally every other day for 12 weeks before sacrifice with cardiac puncture under isoflurane anesthesia. At 3 days before sacrifice, mice were tested for fasting blood glucose (FBG), fasting blood fructose, fasting insulin, homeostatic model evaluation of insulin resistance (HOMA-IR or HOMA index), lipid profile (cholesterol and triglyceride), oral glucose tolerance test (OGTT), insulin tolerance test (ITT), and gut leakage. At sacrifice, the liver and skin were snap frozen in liquid nitrogen and kept at −80 °C before use. Feces from all parts of the colon were combined and collected for microbiome analysis. In particular, the separation of mice was performed here because the microbiome analysis of the same cage could be similar to that of coprophagy (the consumption of feces from other mice). 

### 2.2. Mouse Sample Analysis and Gut Leakage Measurement

After fasting for 12 h with free access to drinking water, fasting blood glucose, fructose, and insulin (FI) were determined by colorimetric assay (glucose and fructose) (Cayman Chemical, Ann Arbor, MI, USA) and mouse Ins1/insulin ELISA kit (Sigma, MO, USA). Total cholesterol and triglycerides were evaluated using a cholesterol and triglyceride quantification kit (Sigma-Aldrich, St. Louis, MO, USA), while low- and high-density lipoprotein cholesterol (LDL and HDL) was quantified by lipid profile assays (Crystal Chem Inc., Downers Grove, IL, USA). Liver damage (serum alanine transaminase) and serum cytokines were determined by the EnzyChrom™ Alanine Transaminase Assay Kit (EALT-100; BioAssay Systems, Hayward, CA, USA) and enzyme-linked immunosorbent assays (ELISA) for mouse cytokines (Invitrogen, Carlsbad, CA, USA), respectively. The HOMA index was calculated followed by the following formula; HOMA index = [fasting insulin in µU/mL × fasting blood glucose in mmol/L]/22.5. For the oral glucose tolerance test (OGTT), mice fasted overnight (16–18 h) were administered orally with glucose solution (2 mg/kg body weight) before blood glucose measurement. For the insulin tolerance test (ITT), fasting mice for 6 h were administered intraperitoneally with human insulin (Humulin R, 0.75 units/kg body weight) before measuring blood glucose at 0, 15, 30, 60, 90, and 120 min afterward. The area under the curve (AUC) of OGTT and ITT was calculated by a trapezoidal rule. Gut permeability was determined by the fluorescein isothiocyanate dextran (FITC-dextran) assay and endotoxemia following previous publications [50,51,52]. As such, FITC-dextran, a non-absorbable molecule with 4.4 kDa molecular mass (Sigma-Aldrich, St. Louis, MO, USA) at 12.5 mg per mouse, was administered orally 3 h before detecting FITC-dextran in serum by fluorospectrometer (NanoDrop 3300; ThermoFisher Scientific, Wilmington, DE, USA). Serum endotoxin (LPS) was measured by detection of HEK-Blue LPS (InvivoGen, San Diego, CA, USA) and data were recorded as 0 when LPS values were less than 0.01 EU/ml due to the limited lower range of the standard curve.

### 2.3. Liver Analysis

For histology, paraffin embedded sections (4 µm thick) stained with hematoxylin and eosin (H & E) of 10% formalin-fixed samples were evaluated. The obesity-induced liver damage scoring system was used as follows; steatosis (0–3), lobular inflammation (0–3), and hepatocellular balloon degeneration (0–2) [53]. The thickness of subcutaneous fat was determined following a previous publication [54]. For the detection of lipids in the liver, the livers were sonicated (High-Intensity Ultrasonic Processor, Newtown, CT, USA) in 500 µL of ice-cold PBS containing the protease inhibitor cocktail (I3786) (Sigma-Aldrich, St. Louis, MO, USA) and lipids were measured from the supernatant by quantification assays as mentioned above. Furthermore, oxidative stress and an antioxidant molecule in the homogenized liver were evaluated after a previous study [55] using malondialdehyde (MDA) and glutathione (GSH) assays (Cayman Chemical Company, Ann Arbor, MI, USA). Furthermore, for cytokine detection in colon tissue, samples were weighed, cut, and thoroughly sonicated (High-Intensity Ultrasonic Processor, Newtown, CT, USA) in 500 mL of ice-cold PBS containing the protease inhibitor Cocktail (I3786; Sigma-Aldrich, St. Louis, MO, USA) and cytokines of the supernatant measured by ELISA (Invitrogen; ThermoFisher Scientific, Wilmington, DE, USA).

### 2.4. Fecal Microbiome Analysis

Feces (0.25 g per mouse) of different cages were used in each experimental group for microbiota analysis following a previous protocol [56]. In summary, metagenomic DNA was extracted from 0.25 g of feces using the DNeasy PowerSoil Kit (Qiagen, MD, USA) using the Miseq300 platform (Illumina, San Diego, CA, USA) at the Omic Sciences and Bioinformatics Centre and Microbiome Research Unit for Probiotics in Food and Cosmetics, Chulalongkorn University. The raw sequences were quality processed and classified into operational taxonomic units (OTU) following Mothur’s standard operating platform procedures [57,58]. Bioinformatic analyses included good coverage, alpha diversity (e.g., Chao), and beta diversity. Linear discriminant effect size analysis (LEfSe) and meta-stats were also performed to determine the species marker and the unique representative species of the interested group, respectively [57,59].

### 2.5. Responses of Enterocytes and Hepatocytes

Caco-2 (HTB-37) or HepG2 (HB-8065) from the American Type Culture Collection (ATCC) (Manassas, VA, USA) was maintained in supplemented Dulbecco’s modified Eagle medium (DMEM) consisting of 5.5 mM glucose at 37 °C under 5% CO_2_ and subcultured before use in the experiments. Cells at 1 × 10^6^ cells/well were then incubated with an additional 25 mM/well of glucose or fructose with or without 100 µg/mL of lipopolysaccharide (LPS) from *E. coli* O26: B6 (Sigma-Aldrich, St. Louis, MO, USA) before determining supernatant cytokines (Quantikine Immunoassay; R & D Systems, Minneapolis, MN, USA). For enterocytes, the expression of occludin (intestinal tight junction) and the nuclear factor kappa B (*NF-κB*) in relative to *β-actin* (a housekeeping gene) was carried out according to the 2^−ΔΔCp^ method [60]. The primers were as follows; *occludin*, forward 5′-CCAATGTCGAGGAGTGGG-3′, reverse 5′-CGCTGCTGTAACGAGGCT-3′; *NF-κB*, forward 5′-AGCACAGATACCACCAAGACC-3′, reverse 3′-GGGCACGATTGTCAAAGAT-5′; *β-actin* forward 5′-CCTGGCACCCAGCACAAT-3′, reverse 5′-GCCGATCCACACGGAGTACT-3′. The monolayer enterocytes were determined by transepithelial electrical resistance (TEER) using Caco-2 cells (HTB-37) at 5 × 10^4^ cells per in the upper compartment of a Tran swell plate from a 24 well chamber for 15 days to establish the confluent monolayer. Subsequently, glucose or fructose (25 mM) with or without 1 µg/mL of LPS LPS) from *E. coli* O26: B6 (Sigma-Aldrich) was incubated at 37 °C at 5% CO_2_. Subsequently, TEER was measured with an epithelial volt ohm meter (EVOM-2, World Precision Instruments, Sarasota, FL, USA) by placing the electrodes in the supernatant at the basolateral and apical chambers. The TEER value in the cell-free medium culture was used as a blank and was subtracted from all measurements. The unit of TEER was ohm (Ω) × cm^2^. The lower TEER represents the higher severity of the Caco-2 cell permeability defect.

### 2.6. Extracellular Flux Analysis

Extracellular flux analysis was performed using Seahorse XFp analyzers (Agilent, Santa Clara, CA, USA) with the oxygen consumption rate (OCR) and the extracellular acidification rate (ECAR) representing mitochondrial function (respiration) and glycolysis activity, respectively, as previously described [23,24,61]. Briefly, stimulated Caco2 or HepG2 cells at 1 × 10^4^ cells/well were incubated with Seahorse medium (DMEM complemented with glucose, pyruvate, and L-glutamine) (Agilent, 103575-100) for 1 h before activation by different metabolic interference compounds, including oligomycin, carbonyl cyanide-4-(trifluoromethoxy)-phenylhydrazone (FCCP) and rotenone/antimycin A, for evaluation of OCR. Parallelly, glycolysis stress tests were performed using glucose, oligomycin, and 2-Deoxy-d-glucose (2-DG) for ECAR measurement. Data were analyzed using Seahorse Wave 2.6 software.

### 2.7. Statistical Analysis

Mean ± standard error (SE) was used for the presentation of the data. The differences between groups were examined for statistical significance by one-way analysis of variance (ANOVA) followed by Tukey’s analysis or Student’s *t* test for comparisons of multiple groups or two groups, respectively. All statistical analyses were performed with SPSS 11.5 software (SPSS, Chicago, IL, USA) and Graph Pad Prism version 7.0 software (La Jolla, CA, USA). A *p*-value of < 0.05 was considered statistically significant.

## 3. Results

### 3.1. Both Glucose and Fructose Caused Obesity-Induced Prediabetes in Mice Attenuated by Lactiplantibacillus plantarum dfa1

**Figure 1:**
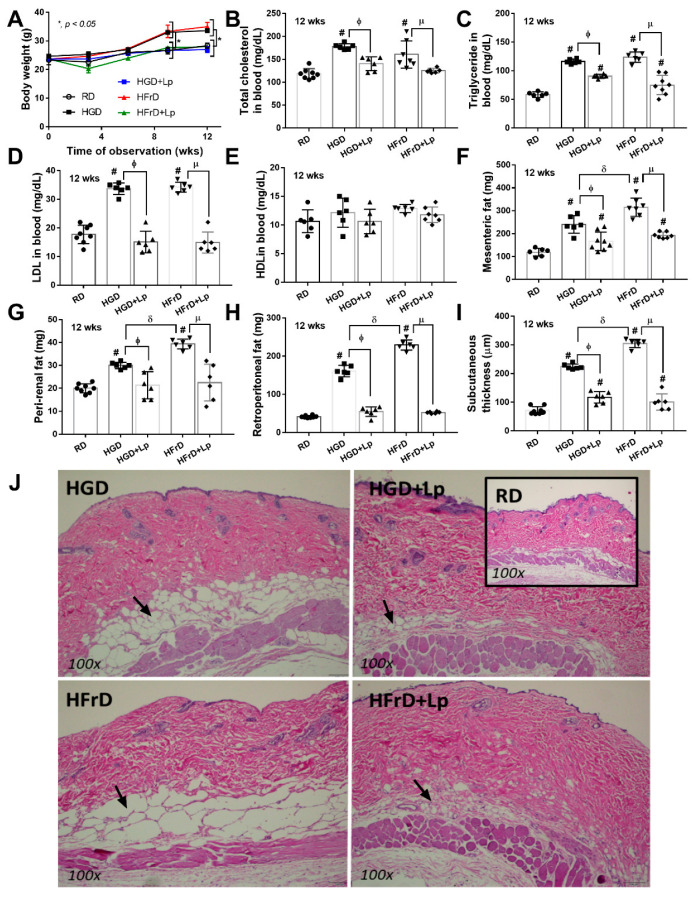
Characteristics of mice with regular diet (RD), or high-glucose diet (HGD), or high-fructose diet (HFrD) with or without *Lactiplantibacillus plantarum* (Lp) are demonstrated as determined by body weight (**A**), fasting blood lipid profile, including total cholesterol, triglyceride, low density lipoprotein (LDL), and high density lipoprotein (HDL) (**B**–**E**), adipose tissue depots in several sites (**F**–**H**), subcutaneous fat thickness (**I**) and representative figures of subcutaneous fat thickness (original magnification 100×) (**J**) are demonstrated (n = 6–8/time point or group). *, *p < 0.05* between indicated groups; #, *p < 0.05* vs. RD; ϕ, *p < 0.05* HGD vs. HGD+Lp; µ, *p < 0.05* HFrD vs. HFrD+Lp; δ, *p < 0.05* HGD vs. HFrD; Arrows indicated subcutaneous fat layers.

**Figure 2:**
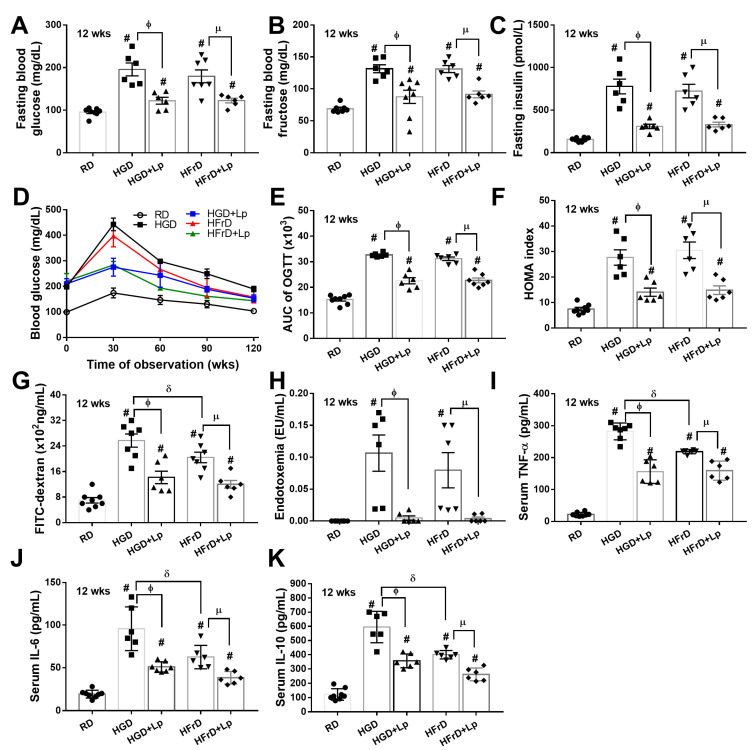
Characteristics of mice with regular diet (RD), or high-glucose diet (HGD), or high-fructose diet (HFrD) with or without *Lactiplantibacillus plantarum* (Lp) are demonstrated as determined by fasting blood profile (glucose, fructose, and insulin) (**A**–**C**), oral glucose tolerance test (OGTT) with the area under the curve (AUC) of the graph (**D**,**E**), homeostatic model evaluation of insulin resistance (HOMA index) (**F**), intestinal leakage (FITC-dextran) (**G**), serum endotoxin (**H**) and serum cytokines (TNF-α, IL-6 and IL-10) (**I**–**K**) (n = 6–8/group or time-point). #, *p < 0.05* vs. RD; ϕ, *p < 0.05* HGD vs. HGD+Lp; µ, *p < 0.05* HFrD vs. HFrD+Lp; δ, *p < 0.05* HGD vs. HFrD.

**Figure 3:**
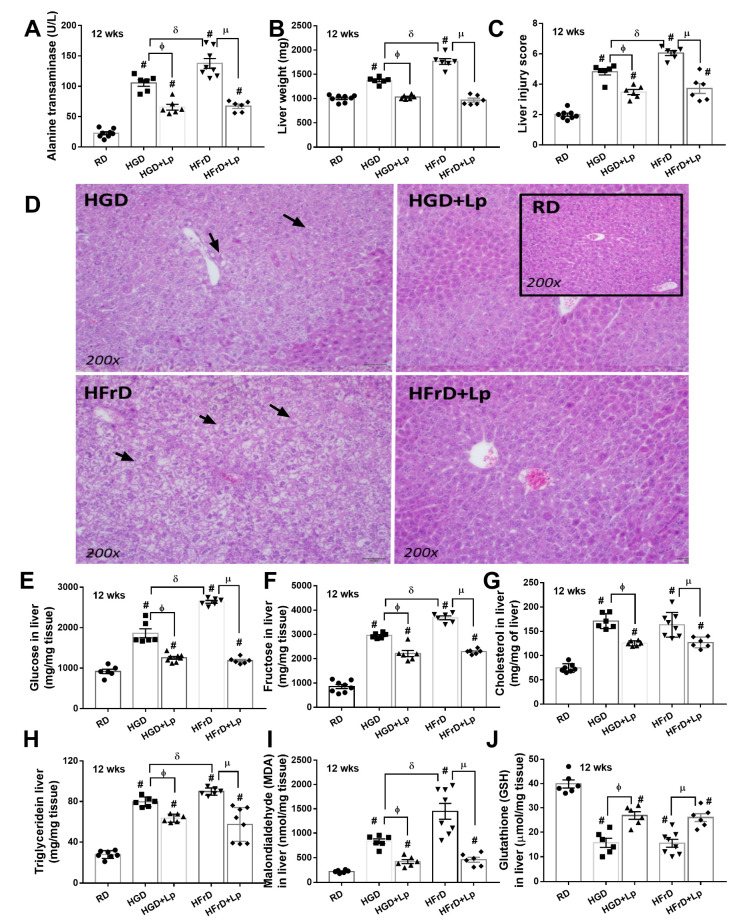
Characteristics of liver injury in mice with a regular diet (RD) or a high-glucose diet (HGD) or fructose diet (HFrD) with or without *Lactiplantibacillus plantarum* (Lp) are demonstrated as determined by alanine (**A**), liver weight (**B**), liver injury score with representative histological pictures (**C**,**D**), liver (glucose, fructose, cholesterol and triglyceride) (**E**–**H**), oxidative stress in the liver (malondialdehyde; MDA) (**I**), and an antioxidant molecule in the liver (glutathione; GSH) (**J**) are demonstrated (n = 6–8/group). #, *p < 0.05* vs. RD; ϕ, *p < 0.05* HGD vs. HGD+Lp; µ, *p < 0.05* HFrD vs. HFrD+Lp; δ, *p < 0.05* HGD vs. HFrD; Arrows indicate hepatocytes with fat accumulation.

Administration of glucose and fructose induced a similar severity of obesity compared to the use of the regular diet, as indicated by body weight (Figure 1A), despite an equal energy content between the high carbohydrate and the regular diet. Significant weight gain was demonstrated at as early as 12 weeks of the experiments (Figure 1A). Similarly, similar obesity parameters, including serum lipid profile (total cholesterol, triglycerides, LDL, and HCL), visceral fat deposition at several sites (mesentery, perirenal, and retro-peritoneum), and subcutaneous fat, were also demonstrated between a diet containing glucose and fructose (Figure 1B–J). Due to well-known obesity-induced prediabetes and insulin resistance [62], several diabetic parameters were determined. As such, a similar severity of prediabetes was demonstrated between glucose and fructose obesity by fasting blood glucose, fructose, and insulin, together with an oral glucose tolerance test and the HOMA index (see method) (Figure 2A–F). In particular, an elevation of fasting blood glucose after fructose administration and vice versa supported the interchangeability between glucose and fructose [63]. Glucose appears to induce more damage to the intestinal tract than fructose, as demonstrated by increased intestinal permeability (FITC-dextran assay); however, with similar endotoxemia (Figure 2G,H). Furthermore, intestinal integrity damage resulted in part in higher serum cytokines (TNF-α, IL-6, and IL-10) in mice administered glucose than fructose administration (Figure 2I–K). On the other hand, the impacts of fructose on the liver appear to be more prominent than those of glucose, at least in part, due to the more rapid intestinal absorption and liver delivery of fructose than glucose [64]. As such, liver damage, as indicated by liver enzyme (alanine transaminase), liver weight, liver injury score from the histology of fructose-administered mice, was higher than glucose administration (Figure 3A–D). The delivery of fructose from the intestinal tract to the liver was possibly better than glucose, as the carbohydrate content (glucose and fructose) in mouse livers after fructose administration was more prominent than in the glucose group (Figure 3E,F), which also supports the interchangeability of glucose and fructose. For liver lipid content, triglycerides, but not cholesterol, alone with oxidative stress (malondialdehyde; MDA) in mouse livers after fructose administration was more prominent than in glucose consumption groups without a difference in the reducing molecule (glutathione; GSH) in livers (Figure 3G–J). These data supported the well-known fructose-induced steatohepatitis and liver injury [65]. 

With *L. plantarum* dfa1 on a high-carbohydrate diet, the body weight of the mouse was not different from that of regular diet control mice that supported the impacts of the antiobesity effects of lactobacilli as previously published [46]. Although fasting blood triglyceride and fat deposition in the mesentery and subcutaneous tissue of probiotic-administered mice were higher than those of the control group, other obesity parameters, including lipid profiles in blood, perirenal fat, and retroperitoneal adipose tissue, were similar to those of control mice (Figure 1B–J), which implies the efficacy of probiotics against obesity. Due to obesity-induced prediabetes [46], the attenuation of weight gain by probiotics reduced the severity of glucose intolerance and insulin resistance, as indicated by several markers (fasting blood glucose, fructose, insulin with OGTT, and HOMA score) (Figure 2A–F). Parallelly, *L. plantarum* dfa1 also strengthened intestinal integrity in obese mice, as evaluated by the FITC-dextran assay, resulting in less severe endotoxemia in conjunction with reduced systemic inflammatory cytokines (Figure 2G–K). Although prediabetic condition, as indicated by higher fasting blood biomarkers, OGTT and HOMA score, and systemic inflammation (serum cytokines) in obese mice administered probiotics with probiotics was still higher than in non-obese control mice, all these parameters improved significantly (Figure 2A–K). Furthermore, *L. plantarum* dfa1 also attenuated liver injury induced by the high-carbohydrate diet [66] as all parameters of liver damage, including liver enzyme, liver histology, liver carbohydrate (glucose and fructose), lipid content, and oxidative stress (MDA) were reduced by probiotics (Figure 3A–I). Despite the more prominent severity of liver injury in fructose administration compared to glucose, probiotics similarly attenuated liver injury in the model (Figure 3A–J). Because (i) Gram-negative bacteria in the gut is a source of endotoxin (LPS) [20] that could enter the bloodstream (obesity-induced endotoxemia) [24] and (ii) intestinal dysbiosis causes a defect of the intestinal barrier [56,67,68], the effect of *L. plantarum* dfa1 on strengthening the intestinal barrier (serum FITC-dextran assay) could be, in part, due to the impact on intestinal dysbiosis.

### 3.2. A Similarity of the Intestinal Microbiota in Mice with Glucose Versus Fructose Administration and a Subtle Impact of Lactiplantibacillus plantarum

**Figure 4:**
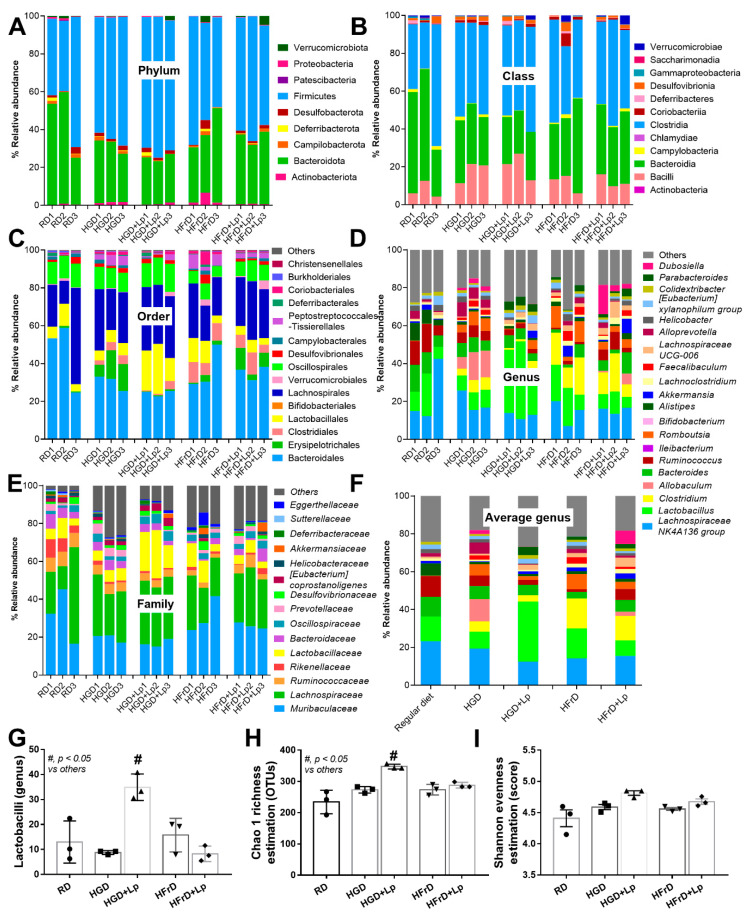
Gut microbiota analysis from feces of mice with regular diet (RD) or high-glucose diet (HGD) or high-fructose diet (HFrD) with or without *Lactiplantibacillus plantarum* (Lp) as determined by the relative abundance of bacterial diversity at the phylum, class, order, family, genus, and average at genus level (**A**–**F**), the graph presentation of Lactobacilli (in genus level) (**G**), and the alpha diversity are demonstrated by estimation of Chao 1 richness and Shannon evenness analysis (**H**,**I**) are demonstrated.

**Figure 5:**
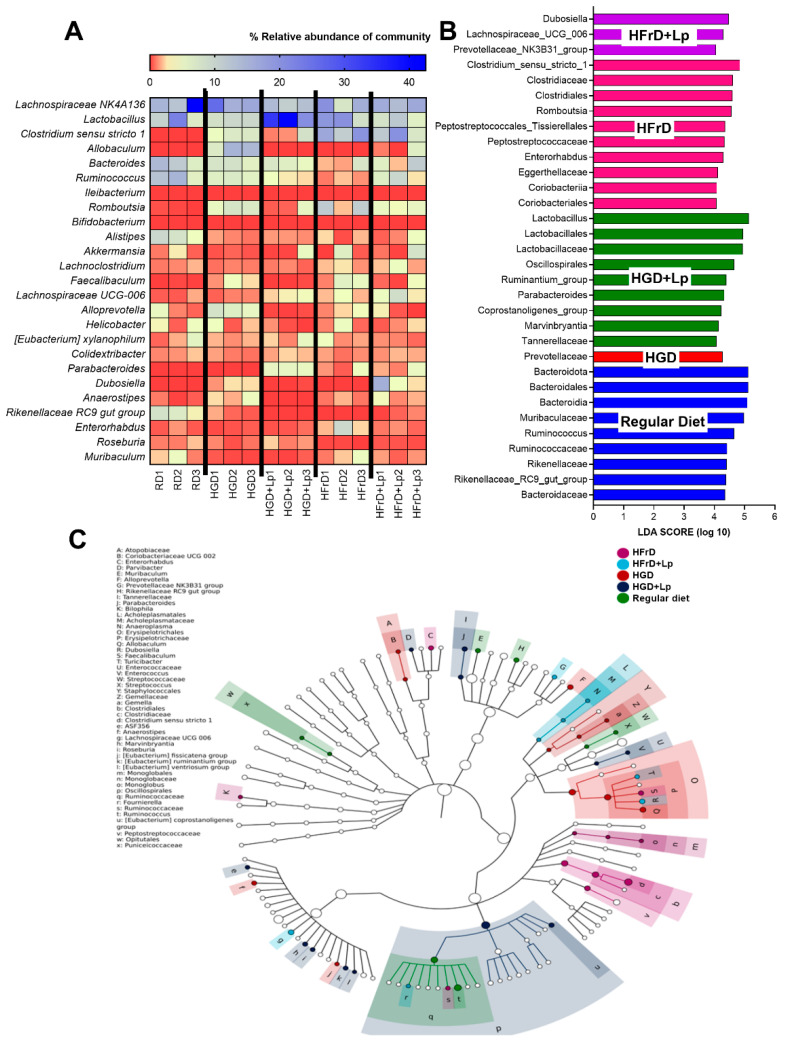
Gut microbiota analysis from feces of mice with regular diet (RD) or high glucose diet (HGD) or high fructose diet (HFrD) with or without *Lactiplantibacillus plantarum* (Lp) as determined by the heat map of relative abundance bacteria (**A**), the possibly unique bacteria in each group by linear discriminant effect size analysis (LEfSe) (**B**), and cladogram (a phylogenetic tree diagram showing the relationships among a group of clades) (**C**) is demonstrated.

There was no difference in the analysis of the fecal microbiome between control non-obese mice and mice with carbohydrate-induced obesity, as indicated by the abundance of bacteria in phylum, class, order, family, genus, and alpha diversity (Chao1 and Shannon score) (Figure 4A–I). However, some differences between mice with high-carbohydrate (glucose or fructose) and regular diet were demonstrated. As such, *Clostridium* bacteria (obligate Gram-positive anaerobe) in the Firmicutes phylum [69] in glucose and fructose group and lower *Allobaculum* spp. (strictly anaerobic Gram-positive bacteria in the Firmicutes phylum [70]) in the fructose group compared to the control were demonstrated (Figure 5A). With the Linear discriminant effect size analysis (LEfSe), the unique bacteria in the control and high-glucose were *Bacteroides* (mostly Gram-negative anaerobes) and *Prevotellaceae* (a group of bacteria in the *Bacteroidota* phylum), respectively, while fructose diet induced *Clostridial* bacteria (Figure 5B). Likewise, the Cladogram (a phylo-genetic tree diagram showing the relationships among a group of clades) were different (Figure 5C). Despite similar Gram-negative bacterial burdens in feces and the fecal microbiota (Figure 4A–I) of mice with a high-carbohydrate diet compared to the regular diet, endotoxemia induced by high carbohydrate diet-induced endotoxemia (Figure 2H) implies an impact of carbohydrate on intestinal permeability [13]. With the administration of probiotics, there was only a subtle change in the analysis of the fecal microbiome. Although *L. plantarum* dfa1 did not alter the fecal abundance of the main group of fecal microbiota, including *Firmicutes* (*Bacillota*), *Bacteroides*, and *Proteobacteria*, probiotic administration increased the estimation of *Lactobacilli* and Chao1 abundance in mice administered glucose, but not in the fructose group, compared to control mice from control mice of regular diet (Figure 4G,H). With the administration of *L. plantarum* dfa1, the representative bacteria in the high glycemic and fructose diet were *Lactobacilli* spp. (the beneficial bacteria) with *Oscillospiraceae* (the normal microbiota in the *Firmicutes* group) and *Duboseilla* spp. (*Firmicutes* group) with *Lachnospiraceae* (*Firmicutes* group), respectively (Figure 5B). Here, the impacts of the high-carbohydrate-diet on the gut microbiota were subtle and resulted in the undetectable beneficial effect of probiotics on gut bacteria alteration.

### 3.3. More Prominent Impact on Glucose Lipopolysaccharide-Induced Enterocyte Damage Than Fructose, with Similar Glucose-Fructose Impacts on Hepatocytes 

**Figure 6:**
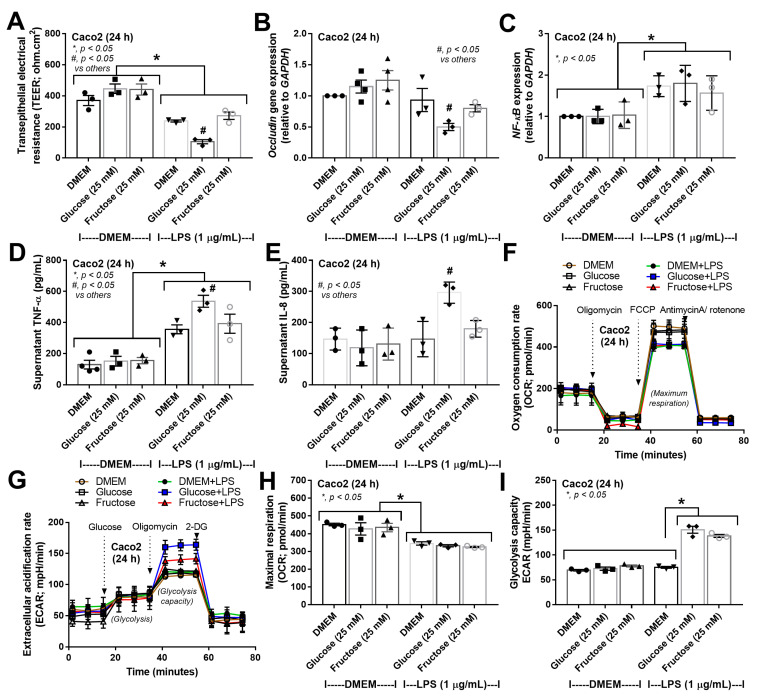
Characteristics of Caco-2 cells (enterocytes) after 24 h of activation by medium control (DMEM), glucose or fructose with and without lipopolysaccharide (LPS) as indicated by transepithelial electrical resistance (TEER) (**A**), *occludin* gene expression (a tight junction molecule) and nuclear factor kappa B (*NF-κB;* a transcription factor) (**B**,**C**), supernatant cytokines (TNF-α and IL-8) (**D**,**E**), the extracellular flux analysis of mitochondrial function and glycolysis activity through oxygen consumption rate (OCR) and extracellular acidification rate (ECAR), respectively, (**F**,**G**), and the graph presentation of maximal respiration from OCR (**H**), and glycolysis capacity of ECAR (**I**) are demonstrated. Independent triplicate experiments were performed for all experiments.

Although the high abundance of lipopolysaccharide (LPS) from Gram-negative bacteria, the most abundant organisms in the intestinal tract, leads to natural resistance of enterocytic responses to LPS [71], a high concentration of LPS in the presence of elevated levels of carbohydrates could have some impact on enterocytes. In measuring enterocyte integrity, a high concentration of glucose and fructose alone did not alter enterocyte permeability measured by transepithelial electrical resistance (TEER), while a high abundance of LPS alone induced enterocyte damage, as indicated by the reduced TEER value (Figure 6A). Furthermore, TEER was lower in LPS plus carbohydrate (glucose or fructose) when compared to LPS alone and LPS with glucose produced more prominent damage than LPS with fructose (Figure 6A), supporting the well-known glucose-induced enterocyte integrity defect [13]. In fact, *occludin* gene expression (an enterocyte tight junction molecule) was negatively regulated by glucose plus LPS, but not under other conditions (Figure 6B). Parallelly, high concentrations of glucose or fructose alone did not induce inflammatory responses, as indicated by the expression of *NF-κB* and supernatant cytokines (TNF-α and IL-8) (Figure 6C–E). However, LPS with glucose, but not fructose, elevated supernatant cytokines (Figure 6C–E), implying a possible higher toxicity of glucose than fructose as a synergy to LPS-induced enterocyte injury. In cell energy status, glucose and fructose alone did not alter mitochondrial and glycolysis activities in Caco-2 cells (Figure 6F–I). However, LPS alone at high doses decreased maximal respiration without alteration of glycolysis activity compared to the DMEM control (Figure 6H,I). However, LPS plus high glucose concentration induced a higher maximal glycolysis than other groups (Figure 6H), possibly correlated with the most prominent enterocyte damage (TEER, *occludin* expression, and inflammatory markers) in this group (Figure 6A–E). Meanwhile, LPS plus glucose or fructose similarly reduced mitochondrial activity (maximal respiration) compared to the DMEM control (Figure 6I). 

**Figure 7:**
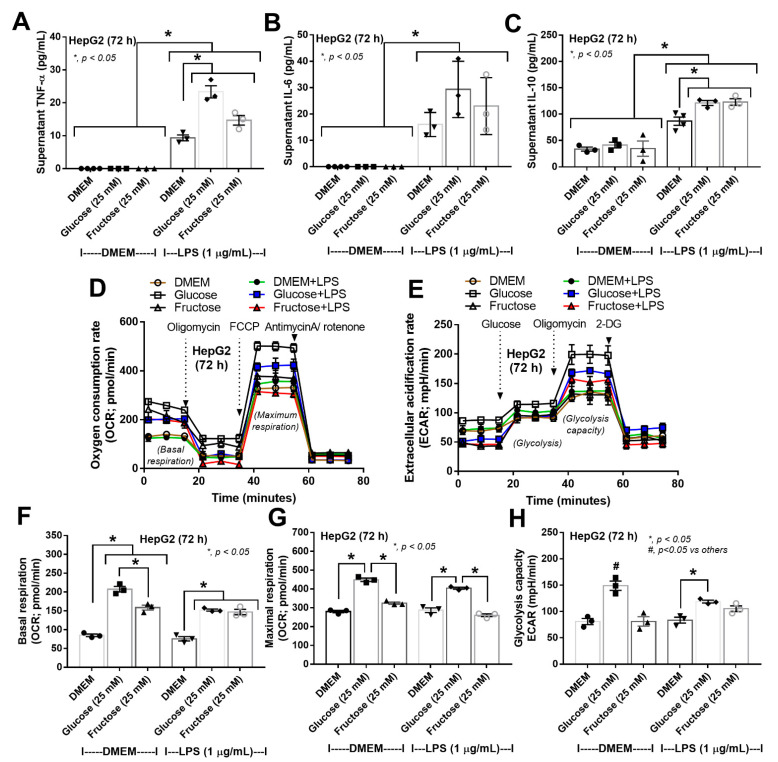
Characteristics of HepG2-2 cells (hepatocytes) after 72 h of activation by medium control (DMEM), glucose or fructose with and without lipopolysaccharide (LPS) as indicated by supernatant cytokines (TNF-α, IL-6, and IL-10) (**A**–**C**), extracellular flux analysis of mitochondrial function and extracellular acidification rate (ECAR) and, respectively, (**D**–**F**) and graph presentation of maximal respiration of OCR (**G**) and glycolysis capacity of ECAR (**H**) are demonstrated. Independent triplicate experiments were performed for all experiments.

For hepatocytes, carbohydrate alone (without LPS) did not induce inflammatory responses (Figure 7A–C), similar to the neutral effect of carbohydrate alone in Caco2 cells (Figure 6A–E). However, 72 h of glucose incubation alone, but not fructose alone, elevated basal respiration and maximal respiration without alteration of glycolysis (Figure 7D–H). In hepatocytes with LPS plus carbohydrate, there was an additive pro-inflammatory effect compared to LPS alone, as demonstrated by supernatant cytokines, without the difference between LPS plus glucose and LPS plus fructose (Figure 7A–C). For cell energy status, LPS alone did not change mitochondrial and glycolysis activities compared to the control with elevated maximal respiration and glycolysis capacity in glucose plus LPS compared to LPS alone (Figure 7D–H). These data suggested various impacts of glucose and fructose on different cell types. 

## 4. Discussion

Although both glucose and fructose induced similar obesity severity, glucose caused more severe leaky gut-induced systemic inflammation, while fructose generated more severe steatohepatitis, and *L. plantarum* dfa1 attenuated all mouse parameters.

### 4.1. Prediabetes with Prominent Steatohepatitis or Systemic Inflammation in Obese Mice after Glucose or Fructose Administration, Respectively, and the Various Effects of Different Sugars

Despite the same calculated energy content at 3.04 kcal/g between high-sugar diets (66.5% carbohydrates with 24.8% glucose or fructose) versus regular diet with 55.5% carbohydrates without sugar component, both diets containing glucose and fructose generated similar obesity in mice. Because (i) the high-sugar diets (both glucose and fructose) consisted of a lower fat component (10% fat) than the regular diet (13.2% fat), and (ii) the daily weight reduction in mouse food was similar in all groups, obesity in experimental mice was the result of sugars but not of the fat component or different amounts of diet consumption. Interestingly, both fructose and glucose could induce indistinguishable prediabetes in mice, as indicated by fasting plasma glucose and OGTT, possibly due to the similar severity of obesity of both forms of carbohydrates. The interchangeability between glucose and fructose is possible in mice, as indicated by increased fasting blood glucose in mice administered fructose and vice versa. Fructose is absorbed from the intestine through glucose transporters 5 (GLUT 5) and diffuses into the bloodstream primarily through GLUT 2 (and also GLUT 5) independently of sodium absorption and ATP hydrolysis, resulting in massive fructose uptake by the liver [64,72]. Meanwhile, glucose in the intestine is mainly absorbed through the Na+/glucose cotransporter 1 (SGLT1), followed by GLUT2 [73]. For increased blood glucose after fructose ingestion, absorbed fructose is usually altered into glucose in the small intestines and livers as a well-known fructose-induced gluconeogenesis in the liver [63,74] that could be responsible for increased blood glucose after fructose administration in our mice. On the other hand, conversion from glucose to fructose in the body is also possible, as endogenous fructose production from absorbed glucose is demonstrated by activating the polyol pathway, which is demonstrated in multiple tissues in the pathogenesis of metabolic syndrome and renal disease [75,76]. The interchangeable glucose-fructose in both fasting blood and liver tissue in our mouse model supports the importance of both sugars in the pathogenesis of metabolic syndrome [77,78,79]. 

Despite the similarity in the severity of prediabetes and obesity, fructose induced steatohepatitis more prominently than glucose in our model. In fact, fructose is metabolized exclusively in the liver by fructokinase, whereas glucose is metabolized anywhere in the body, including the enterocyte before liver transport and is metabolized by liver glucokinase into glucose-6 phosphate and later to fructose-6 phosphate and pyruvate by the rate-limiting enzyme phosphofructokinase [80]. In particular, fructokinase activity is more rapid than glucokinase, as the Michaelis constant (Km), the substrate concentration at which the reaction rate is 50% of the maximal rate (Vmax), of fructokinase (Km 0.5 mM) is much lower than glucokinase (Km 10 mM) indicating a more rapid activation of fructokinase [81]. Furthermore, liver conversion of glucose to fructose-6 phosphate and then to pyruvate is regulated by insulin, while fructose is rapidly transformed directly into triose-phosphate independent of insulin and continuously enters the glycolytic pathway with low Km of fructokinase for fructose, and the absence of negative feedback from ATP or citrate [82]. The largest portion of fructose triose-phosphate is mainly converted to glucose and glycogen through gluconeogenesis, and some parts are converted to lactate [83]. Although the impact of glucose on hepatocytes is controlled by insulin, fructose is converted to fatty acids and improves reesterification of fatty acids and the synthesis of very low-density lipoproteins (VLDL) -triglycerides (TG) without any control systems [84], resulting in higher amounts of lipid in the liver of mice with fructose administration than in the glucose group. In the liver, fructose can also be converted to glucose and glycogen, while glucose is stored as glycogen, and high glucose in hepatocytes (from the ingestion of glucose or fructose) increases the formation of glycerol-3 phosphate and accelerates liver TG production [85]. Therefore, the head-to-head comparison between glucose and fructose ingestion in our model clearly demonstrated the most prominent liver adverse effect of fructose over glucose, especially in terms of the initiation of a nonalcoholic fatty liver and the abundance of lipids in the liver [78]. On the other hand, glucose caused more severe leaky intestinal systemic inflammation (FICT-dextran and serum cytokines) than fructose, despite a similar level of endotoxemia, supporting a possible higher enterocyte toxicity of glucose as mentioned in a previous publication [13]. Despite the well-known intestinal dysbiosis due to the high carbohydrate content of the gut [86], there was only a subtle change between control and carbohydrate-administered mice in our model, perhaps due to differences in fecal collection between different publications. Here, the fecal collection from metabolic cages is a selection of feces from the large intestine, while high carbohydrate could affect the small intestine (the main site of intestinal carbohydrate absorption) [87]. Then the intestinal and liver injury in our model could be mainly due to high carbohydrates themselves, but not to carbohydrate-induced gut dysbiosis. 

### 4.2. Cellular Toxicity of High-Carbohydrate Concentration 

Although it is the main carbohydrate absorption site in the small intestine, hyperglycemia can induce enterocyte injury in all parts of the intestines, as it is one of the leading causes of intestinal integrity damage [88]. Due to the high abundance of LPS in the intestinal contents of the Gram-negative microbiota, the ingestion of carbohydrates in large amounts possibly overcomes the natural resistance to LPS of enterocytes, causing leaky intestinal and systemic inflammation. Similarly, absorbed carbohydrate and pathogen molecules from a leaky intestine are transported early to the liver through the portal vein [20] and the presence of a high abundance of carbohydrate with LPS is also possibly toxic to hepatocytes. Then both enterocytes and hepatocytes were tested with glucose or fructose with or without LPS in vitro. In enterocytes, high concentrations of glucose or fructose alone did not alter enterocyte integrity (TEER) and a high dose of LPS (1 µg/mL) was necessary to reduce TEER (indicating enterocyte tight junction damage) supporting the natural strength of enterocytes against several insults [89]. Due to the need for cell energy for inflammatory responses [90], the LPS-carbohydrate synergy in improving enterocyte inflammation could be due to the increased cell energy of the high carbohydrate content that is ready to be used for LPS-induced cytokine production. In fact, there was an increase in enterocyte maximum glycolysis activity in LPS plus carbohydrate (more prominent in glucose than in fructose). However, the maximum glycolysis activity was not increased by carbohydrate alone without LPS and LPS alone without carbohydrate, implying the synergy of both factors, including LPS with TLR-4 signaling and enterocyte carbohydrate absorption molecules (SGLT1 and GLUT2) in the increase in glycolysis. Furthermore, the differences in enterocyte impacts of glucose and fructose (more toxicity and glycolysis activity by glucose activation than fructose incubation) might be due in part to differences in cell absorption pathways (GLUT2 for fructose versus SGLT1 and GLUT2 for glucose). More studies on these topics are interesting. For hepatocytes, incubation with glucose alone, but not fructose, increased mitochondrial function (basal and maximal respiration) and glycolysis activity (glycolysis capacity), despite non-cytokine production. Although both glucose and fructose enter hepatocytes through GLUT (GLUT2 for glucose versus GLUT2, GLUT5, and GLUT8 for fructose) [91], the additional intracellular metabolism of fructose and glucose by fructokinase and glucokinase, respectively, could be different, which, at least in part, leads to a different impact of both carbohydrates on hepatocytes. In particular, there were also different impacts of carbohydrate on enterocytes and hepatocytes according to the extracellular flux analysis. With LPS, there was an increase in inflammatory responses (supernatant cytokines) and extracellular flux analysis (mitochondria and glycolysis) in LPS plus carbohydrate (similar between glucose and fructose) compared to LPS alone, implying LPS-carbohydrate synergy on hepatocyte inflammation. Despite the similar inflammatory synergy between LPS-glucose versus LPS-fructose in hepatocytes, the impacts of glucose-induced cell energy status in liver cells were more prominent than those of fructose, suggesting the possibly non-cell energy medicated mechanisms of inflammatory synergy between LPS and fructose that could be different from LPS-glucose. Although more mechanistic studies are needed, our data demonstrate a synergy between LPS and high carbohydrate doses on injury in both enterocytes and hepatocytes, partly through an alteration in cell energy status. 

### 4.3. Lactiplantibacillus Plantarum Attenuated the Severity of Mice with High-Sugar Diets

In our model, both glucose and fructose induced similar obesity-induced prediabetes and intestinal barrier defect (FITC-dextran assay and increased serum LPS) with only a subtle change in the intestinal microbiota compared to control mice. Although high sugar diet-induced gut dysbiosis is a well-known characteristic [28], the slightest changes in the microbiota in our study could be due to the limited sample size in each group. Despite this limitation, the alteration of the gut microbiota in both glucose and fructose-administered mice can be demonstrated here through differences in possibly unique bacteria in each group by linear discriminant effect size analysis (LEfSe). Furthermore, the administration of probiotics in mice with a high glucose diet significantly elevated the estimation of Chao-1 richness (the total number of species observed in the community) highlighting the beneficial effect of probiotics, despite the limited number of samples. More studies with an adequate number of mice in each group are needed for a solid conclusion on the alteration of the microbiome induced by a high-sugar diet. However, probiotics are known to attenuate obesity through various mechanisms [24], including more effective energy use [68], promoted intestinal hormones [92], and reduced lipid absorption in the host [93] with a previously known efficacy of *L. plantarum* dfa1 against lipid-induced intestinal damage [46]. Our current data here supported the impact of probiotics on high sugar diet-induced prediabetes; however, indirectly through the reduced weight gain that was possibly more prominent than the influence on gut dysbiosis. As such, probiotics attenuate the severity of the model in nearly all aspects, despite a subtle change in the microbiota of the model. *L. plantarum* dfa1 growth in the feces of mice with high glucose appears to be better than in the feces with fructose, since there was a higher abundance of *Lactobacillus* spp. by microbiome analysis (Figure 4G) that possibly correlated with the estimate of increased fecal microbiota (Figure 4H) only in the glucose group but not in mice administered fructose. However, a similar attenuation effect of probiotics was observed between mice administered fructose and glucose, despite the different abundances of *Lactobacilli* from microbiome analysis, also indicating that the probiotic effect of our high-sugar diet model should be due to an anti-prediabetes or an anti-obesity effect. In fact, *Lactobacillus*, *Bifidobacterium*, *Clostridium*, and *Akkermansia* are indicated as a bacterial group with beneficial changes in insulin resistance through several possible mechanisms (reduced carbohydrate absorption, improved energy utilization, facilitation of some intestinal enzymes and anti-inflammation) [94,95]. There were a number of limitations due to the study’s “proof-of-concept” characteristics, particularly with regard to the mechanical interpretation of the observed data. It would be interesting to see further research on metagenomic, metabolomic, and functional microbiota studies. Although our data support the use of probiotics for the prevention of high-carbohydrate-induced prediabetes, more research on these subjects is needed for the upcoming clinical translation.

## 5. Conclusions

Although both the high-glycemic and high-fructose diet are harmful, there are possible different health impacts between these sugars. Indeed, glucose caused more prominent damage to intestinal integrity (leaky gut), while fructose caused more profound steatohepatitis. However, both sugars induced a similar severity of prediabetes and obesity. Because LPS is an overwhelming pathogen molecule in the intestine that can be transferred to the liver during leaky intestine, simultaneous stimulation by sugars and LPS in enterocytes and hepatocytes is regularly possible. Interestingly, glucose plus LPS induced a more prominent injury in enterocytes and hepatocytes than fructose with LPS, as indicated by TEER and supernatant cytokines, respectively, partly through a more prominent glycolysis activity. *L. plantarum* dfa1 effectively attenuated prediabetes and obesity despite only a subtle impact on the gut microbiota, which implies a possible impact on insulin resistance. The use of probiotics is encouraged to prevent carbohydrate-induced prediabetes.

## Data Availability

Data is contained within the article.
